# Why Do We Put Cervical Collars On Conscious Trauma Patients?

**DOI:** 10.1186/1757-7241-17-44

**Published:** 2009-09-18

**Authors:** Jonathan Benger, Julian Blackham

**Affiliations:** 1 Professor of Emergency Care, Faculty of Health and Life Sciences, University of the West of England, Bristol, UK; 2 Specialist Trainee in Emergency Medicine, Academic Department of Emergency Care, University Hospitals Bristol NHS Foundation Trust, Bristol, UK

## Abstract

In this commentary we argue that fully alert, stable and co-operative trauma patients do not require the application of a semi-rigid cervical collar, even if they are suspected of underlying cervical spine fracture, unless their conscious level deteriorates or they find the short-term support of a cervical collar helpful. Despite the historical and cultural barriers that exist, the potential benefits are such that this hypothesis merits rigorous testing in well-designed research trials.

## Introduction

"The staff must be continually cognizant that injudicious manipulation or movement, and inadequate immobilisation can cause additional spinal injury and decrease the patient's overall prognosis"

Advanced Trauma Life Support Course Manual, Sixth Edition

The above quote exemplifies an approach to cervical spine management that has prevailed in the developed world for almost three decades. The underlying premise seems intuitively sound, but has been carried to lengths that are now more harmful than helpful to the vast majority of trauma patients. In this commentary we argue that fully alert, stable and co-operative trauma patients do not require the application of a semi-rigid cervical collar, even if they are suspected of underlying cervical spine fracture, unless their conscious level deteriorates or they find the short-term support of a cervical collar helpful. Despite the historical and cultural barriers that exist, the potential benefits are such that this hypothesis merits rigorous testing in well-designed research trials.

## Discussion

Patients with potential cervical spine injury are a common problem for pre-hospital and in-hospital trauma practitioners. Their management is time consuming, complicates extrication and creates a significant workload in immobilisation, transportation and management.

Pre-hospital spinal immobilisation is broadly applied in patients at risk of cervical spine injury. This practice is recommended in resuscitation guidelines such as Advanced Trauma Life Support (ATLS), Pre-Hospital Trauma Life Support (PHTLS) and the Joint Royal Colleges Ambulance Liaison Committee (JRCALC) guidelines. [1-3] However, despite the widespread use of cervical spine immobilisation there is very little evidence that it is beneficial.[4] Furthermore, a number of studies have noted the harm caused by prolonged spinal immobilisation, including decubitus ulcers from lying on hard boards, and increased jugular venous pressure resulting from the application of a semi-rigid cervical collar (see below). Hauswald argued in 1998 that the initial impact will cause injury to the spinal cord, and subsequent movement is very unlikely to cause any further damage.[5]

Since universal immobilisation is rarely beneficial and carries an element of risk as well as inconvenience there is a clear need for guidelines that rationalise the use of immobilisation. During the latter half of the 20^th ^Century clinical decision rules were developed and validated that are able to identify a sub-set of alert and co-operative patients who do not require cervical spine immobilisation or radiography. Chief among these are the NEXUS low risk criteria,[6] and the Canadian C-Spine Rules.[7] Whilst the superiority of one approach over the other is hotly debated,[8] the specificity of both rules is such that they still mandate immobilisation in a large proportion of injured patients.

Every day thousands of alert and co-operative people across Europe have a semi-rigid collar applied to their neck shortly after trauma "as a precaution". They are then usually laid supine, fully immobilised on a long extrication board or similar device and conveyed to hospital, remaining in this inconvenient state for prolonged periods pending clinical assessment and radiological imaging. Yet the overwhelming majority have no spinal injury.

The assumptions that underpin cervical spine immobilisation are as follows:

1. Injured patients may have an unstable injury of the cervical spine.

2. Further movement of the cervical spine could cause additional damage to the spinal cord, over and above that already caused by the initial trauma itself.

3. The application of a semi-rigid cervical collar prevents potentially harmful movements of the cervical spine.

4. Immobilisation of the cervical spine is a relatively harmless measure, and can therefore be applied to a large number of patients with a relatively low risk of injury "as a precaution".

We will address each of these points in turn.

Firstly, there is no doubt that trauma can cause an unstable injury of the cervical spine. We will not further debate this point, except to note that unstable cervical spine injuries in otherwise alert, stable and co-operative patients are rare. The UK incidence of spinal cord trauma is 10-15 per million population per year,[9] with a little more than half of these injuries in the cervical spine.[10,11] In the alert and stable patient cohort studied by Stiell and colleagues the incidence of "clinically important" cervical spine injury was 1.7%, with 0.1% developing a neurological deficit.[7]

Secondly, we turn to the question of whether cervical spine movement in an unstable injury will lead to neurological impairment. It is well documented that neurological signs can progress following spinal cord injury, but the cause of this progression is less clear. Spinal cord haemorrhage and oedema both occur following trauma, and complicate the assessment of further movement as a contributing factor. The progression of injury that was previously noted in some patients and used as a rationale for universal immobilisation is therefore difficult to interpret. Clearly, the initial forces required to create an unstable injury of the cervical spine will be considerable, and it seems unlikely that small degrees of further movement will worsen the situation. In an unconscious patient who is being transferred from one location to another the application of measures to stabilise the head, and therefore reduce the risk of sudden uncontrolled neck movements, seems logical, but what of patients who are already fully in control of their own neck? The natural effects of injury are pain and protective muscle spasm with a marked reluctance to move the injured part: why should the cervical spine be any different?

Thirdly, we should ask whether the application of a semi-rigid collar to an alert and stable patient actually prevents potentially harmful movements of the cervical spine over and above the natural protection afforded by the patient themselves. Cervical collars are known to be poorly applied, and it seems unlikely that a single design will be appropriate for all patients and all possible unstable injuries of the cervical spine. Indeed, everyday observation of patients brought to our Emergency Department in a cervical collar show many in hyper-extension, and others where poor fitting of the collar has led to various degrees of lateral flexion or apparently unrestricted movement. Collars do reduce movement of the neck, but even correctly fitted ones allow over 30° of flexion/extension and rotation.[12] This is improved by the use of sandbags and tape, which on their own provide better cervical spine immobilisation than a collar alone.[13]

Finally, we come to the harms associated with cervical collars, even those applied for only a few hours. Most patients complain that collars are uncomfortable to wear in the short term. There are also case reports of patients whose condition has deteriorated after a cervical collar has been fitted, particularly those with ankylosing spondylitis or rheumatoid arthritis.[14] This may reflect the fact that some patients have existing deformities or fragilities of the cervical spine, and are forced into unfavourable or even harmful positions when a cervical collar is applied.

Cervical spine injury is often suspected in the presence of head injury, but collars significantly increase intracranial pressure: an effect that is even more pronounced when a head injury is actually present.[15] In addition, most patients with suspected spinal injury are transported to hospital on long extrication boards (often called long spinal boards). These are actually designed for extrication, and as a transport device the long board is far from ideal. It has a hard, flat and slippery surface that causes pain in patients who lie on it for any period of time. A study in 1989 found that 21% of patients with cervical spine pain and 33% of patients with lumbar spine pain while immobilised on a long board experienced complete resolution of their symptoms once removed from the board.[16] By measuring interface pressures between the skin and different surfaces in healthy volunteers Main and Lovell showed that the highest pressures are found at the sacrum (233.5 mmHg) and the thorax (82.9 mmHg).[17] Experimental studies have suggested that a constant pressure of 70 mm Hg for more than two hours produces tissue ischaemia and irreversible tissue damage.[18]

Another study by Paterson and colleagues demonstrated that transcutaneous oxygen tensions are significantly lower in patients with spinal injuries (7.3 mmHg vs. 27.2 mmHg) when a 30 mmHg pressure is applied to the anterior tibia.[19] This demonstrates that patients with spinal injuries are more at risk of tissue damage from immobilisation on a hard surface than those without spinal injury. Immobilisation in a supine position also causes a considerable reduction in respiratory function. The functional residual capacity and forced expiratory volume in one second are both reduced, even in healthy, non-smoking volunteers.[20] Given that some patients who are immobilised will also have pre-existing or acute cardio-respiratory disease (e.g. the elderly with chest wall injuries) this will have a clearly detrimental effect. In a small number of patients, particularly those with facial trauma and haemorrhage into the airway, supine immobilisation may even lead to catastrophic airway compromise.

Finally, it is worth noting that transport in an ambulance whilst immobilised in a supine position may precipitate motion sickness, vomiting and even aspiration. This is inconvenient, and potentially harmful. It is also particularly challenging to maintain enforced spinal immobilisation whilst a patient is actively vomiting in a moving ambulance.

In summary, therefore, immobilisation on a long extrication board is uncomfortable, causes neck and back pain, pre-disposes to pressure sores, compromises respiratory function, and may precipitate vomiting. The actual spinal immobilisation achieved is also less than that provided by a vacuum mattress.[21]

Whilst the immobilisation of alert and co-operative patients may appear intuitive, and is strongly based on tradition, it is not supported by a reliable body of evidence. We are unable to find any reports of acute deterioration in an alert and co-operative patient with cervical spine injury as a result of a failure to immobilise shortly after injury. Where an unstable cervical spine injury is initially overlooked in an ambulant patient the natural history appears to be one of gradual deterioration over subsequent weeks and months (presumably as the initial protective muscle spasm subsides) rather than sudden, catastrophic neurological impairment in the first 24 hours. This is supported by evidence from an evaluation of physician performance without the assistance of a clinical decision rule, which identified nine patients (all alert and ambulant) with clinically significant cervical spine injuries who were erroneously discharged from the ED. However none came to subsequent harm.[22] Furthermore, a comparison between a country that operates a protocol of full immobilisation and one that has no immobilisation found no difference in the neurological outcomes of 454 patients with blunt spinal injuries.[5]

For patients unable to protect their own cervical spine (e.g. those with a reduced level of consciousness, or apparently under the influence of alcohol and/or drugs) a policy of immobilisation remains sensible and appropriate. It is also important to ensure adequate spinal protection when a patient's condition deteriorates such that their level of consciousness falls, or their clinical management requires sedation or anaesthesia. However, for the vast majority of trauma patients, who are fully alert, stable and co-operative when their cervical spine is immobilised, we suggest that this is an unnecessary and potentially harmful precaution. Natural muscle spasm will provide protection that is far superior to any artificially imposed or universal posture, and the position that the patient themselves finds most comfortable (the "position of comfort") is likely to be the best for their particular injury. If the patient wishes to lie supine, or finds the support of a collar helpful, then this should be arranged. Otherwise, the most useful function of a collar is as a visible signal that the neck has not yet been fully assessed, and may need radiological imaging. Indeed, were the concept of "position of comfort" to be universally adopted it would be important to find alternative ways of communicating concern regarding potential cervical injury between healthcare professionals.

## Conclusion

In conclusion, we hypothesise that alert, stable and co-operative trauma patients do not require mandatory immobilisation of the cervical spine, even if a clinical decision rule is positive and radiography is indicated. Instead, a "position of comfort" selected by the patient (and including a cervical collar and supine positioning only if found to be beneficial by that individual) may be more appropriate pending further clinical evaluation. This is consistent with our current ED practice, in that patients who are ambulatory and self-present to the ED with possible cervical spine injury are not routinely immobilised, and no case of sudden neurological deterioration has been recorded in this group. We therefore advocate a large-scale research study to test this hypothesis, with considerable potential benefits to the thousands of trauma patients who undergo cervical spine immobilisation worldwide every day.

## Competing interests

The authors declare that they have no competing interests.

## Authors' contributions

Jonathan Benger had the initial idea for this commentary, and drafted the manuscript. Julian Blackham performed the literature search and revised the manuscript. All authors read and approved the final manuscript.
